# Resolution of Null and Overt Pronouns in Catalan: An Eye Tracking Study

**DOI:** 10.1007/s10936-025-10189-7

**Published:** 2026-01-10

**Authors:** Aurora Bel, Ernesto Guerra, Nadia Ahufinger, Llorenç Andreu, Mònica Sanz-Torrent

**Affiliations:** 1 Departament de Traducció i Ciències del Llenguatge, Universitat Pompeu Fabra, Roc Boronat, 138, 08018 Barcelona, Spain; 2 Centro de Investigación Avanzada en Educación, Instituto de Educación, Universidad de Chile, Periodista José Carrasco Tapia 75, 8330014 Santiago, Chile; 3 Departamento de Lingüística, Facultad de Filosofía y Humanidades, Universidad de Chile, Periodista José Carrasco Tapia 75, 8330014 Santiago, Chile; 4 Estudis de Psicologia i Ciències de l’Educació, NeuroDevelop eHealth Lab, eHealth Center, Universitat Oberta de Catalunya, Rambla del Poblenou, 154-156, 08018 Barcelona, Spain; 5 NeuroDevelop eHealth Lab, eHealth Center, Universitat Oberta de Catalunya, Rambla del Poblenou, 154-156, 08018 Barcelona, Spain; 6 Departament de Cognició, Desenvolupament i Psicologia de l’Educació, Secció Cognició, Edifici de Ponent, Universitat de Barcelona, Pg. de la Vall d’Hebron, 171, 08035 Barcelona, Spain

**Keywords:** Pronoun resolution, Order of mention, Word order, Eyetracking, Catalan

## Abstract

This study investigates subject pronoun resolution in Catalan, a null-subject language, by examining linguistic and cognitive factors. We focus on gender, pronoun type (null and overt), and syntactic function (subject and object), as well as the order of mention (first and second) of antecedents. Using eye-tracking in the Visual World Paradigm (VWP), we analyze the interplay between these factors in both canonical (SVO) and non-canonical (OVS) sentence structures. Experiments 1, 2 and 3 (Study 1) explore how gender cues and pronoun type influence pronominal resolution in SVO sentences. Our findings reveal that gender facilitates early resolution but primarily affects first-mentioned antecedents. Null pronouns exhibit a strong preference for subject antecedents. However, overt pronouns, contrary to offline studies, show no strong preference for object antecedents and instead tend to favor first-mentioned subjects in ambiguous contexts. In unambiguous gender-marked cases, overt pronouns heavily rely on gender cues for reference resolution, but in ambiguous contexts, they display delayed resolution patterns. These results suggest that overt and null pronouns do not exhibit the expected division of labor proposed by the Position of Antecedent Hypothesis (PAH). Instead, null pronouns robustly refer to subject antecedents, while overt pronouns show more variability. Experiments 4 and 5 (Study 2) extend this investigation to OVS sentences, revealing that linear order exerts a stronger influence on pronoun resolution than grammatical subjecthood. Specifically, first-mentioned antecedents, rather than syntactic subjects, guide resolution in OVS structures, particularly for overt pronouns. This challenges previous findings that overt pronouns prefer object antecedents and highlights positional prominence as a crucial factor in pronoun resolution in Catalan. Overall, our results contribute to a broader understanding of pronoun processing across null-subject languages and underscore the importance of positional prominence in pronominal interpretation and suggest that, in non-canonical structures, order-of-mention can override subjecthood in guiding resolution.

**Supplementary Information:**

The online version contains supplementary material available at 10.1007/s10936-025-10189-7.

## Introduction

Effective communication in language relies not just on what is said, but on how efficiently information is conveyed. To achieve this, language users employ different linguistic strategies to reduce redundancy and maintain the flow of discourse. One key strategy involves the use of different referring expressions to point to the same discourse referents without losing coherence. From the listener’s or reader’s standpoint, understanding these connections involves resolving anaphoric dependencies—linking expressions in the text that refer to the same entity or idea. Pronouns, in particular, pose a unique challenge as they are ‘underspecified’ referring expressions that typically require antecedents. Their interpretation depends on identifying the correct antecedent, a process influenced by factors such as the saliency or prominence of potential referents (e.g., Ariel, 1990, among many others).

In this paper, we are interested in analyzing how the speakers of a language interpret different (null and overt) pronominal elements that co-refer with previously mentioned elements (backward anaphora) to decode the message and to provide a complete meaning. We concentrate on third person personal pronouns in subject position, which in languages like Catalan, or Spanish, can be either null or overt. In contrast to first and second person pronouns that respectively refer to the speaker or the addressee, third-person pronouns are anaphoric in nature meaning their referential content relies on the antecedent they co-refer with. Although third-person pronouns can also obtain reference deictically, pointing to elements in the situational context, this study does not address that aspect.

The study of anaphoric dependency resolution and pronoun interpretation, particularly in ambiguous contexts, has been a significant focus in linguistic and psycholinguistic studies. Research has identified different linguistic and cognitive factors that impact the identification of antecedents (see Arnold, 2010; Holler & Suckow, 2016, for a review). Key factors that make antecedents more prominent or accessible include the type of sentential relation, the way clauses are linked and ordered, and the structural position of the antecedent (whether it is a subject or non-subject, or its order of mention). Additionally, the type of anaphor used (such as null or overt pronouns in languages that allow both, demonstratives, etc.) plays a critical role in interpretation, as outlined in Kaiser and Trueswell’s (2008) form-specific approach. The informational status of the antecedent (whether it is a topic or non-topic) is another influencing factor, highlighted as a marker of prominence that enhances attention and accessibility in memory retrieval (e.g., Arnold et al., 2013; Blything et al., 2021, among others). However, there is still no consensus on which factors are most influential in determining pronoun reference, or whether these factors can be systematically ranked in order of importance.

In the current research, we focus on intersentential contexts with two potential antecedent candidates occupying different structural positions (subject vs. object), examining both referentially ambiguous (same-gender antecedents) and non-ambiguous (different-gender antecedents) scenarios. Our primary goal is to investigate whether semantic factors, such as gender; syntactic factors, like grammatical function (or order of mention, as discussed later); and pragmatic factors, such as those derived from canonical and non-canonical word order that affect information status, act as reliable cues in guiding the interpretation of intersentential pronominal anaphora in Catalan. Additionally, we aim to explore how these cues impact the resolution of both null and overt pronouns and the extent to which their effects vary depending on the pronoun type. To address this, we designed five complementary experiments, each targeting one of these factors. Given that Catalan—like Spanish and Italian—includes both null and overt subject pronouns, allows for manipulation of word order, and remains relatively unexplored in this domain, we have chosen to focus specifically on this language.

### Linguistic and Cognitive Factors in Pronominal Anaphora Resolution

Numerous studies have shown that speakers and listeners establish coreference and resolve pronominal anaphora quickly in real time (e.g., Arnold et al., 2000; Arnold, 2010; Dahan et al., 2002). However, this efficiency does not imply that selecting appropriate antecedents and forming coreferent chains is a simple or arbitrary process. Theories like *Accessibility Theory* (Ariel, 1990, 2001) and *Centering Theory* (Grosz et al., 1995) suggest that anaphora resolution is a guided, semi-automatic process shaped by cognitive and discourse-level principles. *Accessibility Theory* posits that the salience of referring expressions influences their likelihood of being chosen as antecedents: the more reduced a referential expression (e.g., a pronoun), the more accessible the antecedent it selects (e.g., a noun phrase). In contrast, *Centering Theory* emphasizes two main claims: (1) the syntactic function of elements determines their prominence in discourse, and (2) pronouns help maintain discourse coherence by linking to highly prominent referents. These ideas overlap with those in *Accessibility Theory*, highlighting the importance of antecedent salience. Originally focused on English overt pronouns, *Centering Theory* was later adapted for null subject languages, revealing that null pronouns are preferred for topic continuity, while overt pronouns signal topic shifts. Briefly, the basic idea which these two proposals share is that the process of establishing coreference arises from the accessibility of the potential antecedents and the factors that enhance discourse prominence (von Heusinger & Shumacher 2019).

In the last two decades, several studies have examined the role of different sources of information in determining the accessibility of an antecedent in order to solve anaphoric dependencies, yielding mixed results. These factors fall into three main categories: semantic (e.g., implicit causality and gender), structural (e.g., syntactic position), and pragmatic (e.g., discourse status or information structure).

Starting with **semantic** factors in reference attribution, verb **implicit causality** (IC) and antecedent’s gender are often cited to explain variation in pronominal antecedent preferences. IC has strong support from both online and offline evidence, showing that the semantics of interpersonal transitive verbs bias interpretation toward one antecedent (Garvey et al., 1976; Hartshorne et al., 2013). Yet its role in anaphora resolution remains debated. Some studies suggest IC affects pronoun resolution only during sentence-final integration (Garnham, 2001; Stewart et al., 2000), whereas others, using visual-world paradigms and ERPs, report that IC biases appear even before disambiguating cues (van Berkum et al., 2007; Järvikivi et al., 2017). Fedele and Kaiser (2014) further show that IC interacts with sentence boundaries, influencing null and overt pronouns in Italian. In our experiments, however, IC effects were neutralized to isolate other linguistic factors.

**Gender**, along with number, plays an important role in pronoun resolution and in determining the prominence of discourse entities that serve as antecedents. However, research on the influence of gender cues in anaphora interpretation has produced mixed results. Offline studies have often identified gender as a key factor in locating a pronoun’s antecedent (Carreiras et al., 1993; Crawley et al., 1990), whereas the *Minimalist Hypothesis* (McKoon & Ratcliff, 1992) emphasizes accessibility over gender cues. Findings from online studies are also inconsistent: while some (e.g., Garnham et al., 1992; McDonald & MacWhinney, 1995) report limited access to gender cues during pronoun interpretation, others (e.g., Cunnings et al., 2017; Hudson-D’Zmura & Tanenhaus, 1998) find evidence for their early influence. Eye-tracking research using the visual-world paradigm likewise shows anticipatory eye movements to gender cues (Arnold et al., 2000, 2007), though these studies also note that in ambiguous contexts—where multiple same-gender antecedents are possible—discourse and structural factors become decisive. In sum, despite extensive investigation, the role of gender in pronoun resolution remains debated.

Notably, Arnold et al. (2000) examined how gender features interact with order of mention in English pronoun resolution through an eye-tracking study. Participants listened to pairs of same-gender ambiguous sentences, where the subject pronoun in the second sentence could potentially refer to either of the two previously introduced characters. In gender-ambiguous contexts, participants initially resolved the pronoun to the first-mentioned character, even when later predicates favored the second. In contrast, in unambiguous different-gender contexts, they quickly identified the gender-matching antecedent. This indicates that both structural cues and gender information affect pronoun processing. A follow-up study with children (Arnold et al., 2007) showed that adults relied more on structural cues, while children prioritized gender information, suggesting differing strategies in coreference resolution. In the present study, we neutralize verb implicit causality across all experiments and incorporate antecedent gender -whether same or different- as a factor in **Experiments 1 and 2**. This approach partially replicates Arnold et al. (2000, 2007) and focuses on overt subject pronouns, allowing for comparability with English overt pronouns.

Continuing with **syntactic** factors, referential interpretation and processing are also sensitive to structural influences. Two structural strategies have received considerable attention: the **First-mention** bias and the **Subject-preference** approach.

A number of studies argued that pronouns tend to establish coreferential chains with first-mentioned syntactic elements of the previous clause or sentence, regardless of their grammatical function (Arnold et al., 2000; Arnold et al., 2018; McDonald & MacWhinney, 1995; Stevenson et al., 1994, among many others). These results support the structure-building framework in sentence processing (see Gernsbacher, 1997 for an overview), which embodies the idea that first mentioned entities constitute the starting point to chart further information as the mental representation of a sentence is being built. Other researchers have provided experimental evidence that a subject pronoun is more likely to be interpreted as referring to the subject of the preceding sentence (Frederiksen, 1981; Song & Fisher, 2005, among others). In a related line of work, Christianson and Cho (2009) examined how null pronouns are interpreted in isolated Korean sentences, showing that even without discourse context, syntactic and semantic cues guide reference assignment. Their findings were broadly consistent with Almor’s Informational Load Hypothesis (Gelormini-Lezama & Almor, 2011), according to which overt pronouns tend to appear when something atypical or contrastive occurs, while null forms are generally preferred otherwise. Findings in Spanish by Carreiras et al. (1993) have also evinced that the first-mention advantage is not absolute but can be modulated by discourse-pragmatic factors such as word order, prominence, and contextual information.

However, subject preference and first mention, although sometimes conflated, represent different strategies. Subject, in contrast to object, clearly refers to a syntactic notion, while first mention, as opposed to second mention, relates to a cognitive strategy that considers linearity. In most of the mentioned research, a significant part of which is focused on English, **order of mention and syntactic function are confounded**. Thus, it is not surprising that it has been defended that these two factors are not exclusive but convergent (Gordon & Chan, 1995). In addition, if a potential antecedent is simultaneously a subject and a topic, thus appearing in first-mention position, its prominence is enhanced and it becomes the best candidate for coreference. Trying to **disentangle syntactic function and (linear) order of mention**, research on languages that present flexible word order like Finnish, a case-marked language, suggests that both order-of-mention and syntactic function have effects on ambiguous pronoun resolution but their role is, to some extent, independent given that subject preference appears earlier than first-mention in that the time-course analyses as measured by eye movement measures (Järvikivi et al., 2005; see, however, Kaiser & Trueswell 2003 for some slightly different results with subject coming out as the stronger factor). Comparable results have been obtained for German (Hert et al., 2024).

Additionally, research on reference tracking has shown that the **type of subject pronoun** is relevant, as different anaphoric forms (such as pronouns and demonstratives) exhibit varying degrees of sensitivity to different factors. Interest has grown in the conditions shaping anaphoric demonstrative interpretation. Kaiser and Trueswell (2008) found that Finnish pronouns and demonstratives select antecedents differently based on grammatical function and position. More recently, Hert et al. (2024) showed that German subject pronouns and demonstratives respond differently to syntactic (e.g., subject) and pragmatic (e.g., focus) factors. Additional evidence comes from Japanese, where null pronouns are sensitive to grammatical function, while overt pronouns align with verb aspect (Ueno & Kehler, 2010).

Null subject languages, having both null and overt subject pronouns, present and additional interest. This set of languages have been extensively studied in offline research, particularly following Carminati’s (2002) *Position of Antecedent Hypothesis* (PAH) for intra-sentential anaphora in Italian. According to the PAH, particularly in ambiguous contexts, null pronouns prefer subject antecedents, while overt pronouns show a bias for object antecedents. Though originally proposed for Italian, this pattern has been observed in other languages, such as Catalan (Bel & García-Alcaraz, 2018; Mayol & Clark, 2010; de Rocafiguera & Bel, 2025), Greek (Papadopoulou et al., 2015), Portuguese (Rinke & Flores, 2018), Spanish (Contemori & Di Domenico, 2021), and Polish (Wolna et al., 2022). However, real-time reference tracking in Romance languages like Spanish, Italian, and Catalan has yet to be fully explored. In **Experiment 3**, we aim to identify nuanced preferences for null subject pronouns in antecedent assignment within ambiguous same-gender contexts, and to compare these preferences with those for overt pronouns observed in Experiment 2.

Finally, **pragmatic** factors such as topic and focus also shape anaphora resolution by influencing the prominence and accessibility of potential antecedents. Manipulating these features creates contrasts between canonical and non-canonical sentence structures, allowing researchers to **disentangle** syntactic roles (subject vs. object) from linear order (first vs. second mention), giving rise to canonical and non-canonical sentence structures with distinct pragmatic meanings. A key device in this domain is **focalization** -often realized through clefting- which highlights specific information and alters the accessibility of referential antecedents. Studies on anaphora resolution with marked structures like *it*-clefts (e.g., “It was Peter who John interrupted when he started speaking,” where *Peter* is the focus) show mixed results. While some findings suggest that linguistic focus increases accessibility of focused entities (Káldi & Babarczy, 2021), focused entities do not consistently serve as preferred antecedents for subject pronouns (Blything et al., 2021). Additionally, clefted structures can lead to an “anti-focus effect,” diminishing accessibility for anaphoric subject pronouns (Colonna et al., 2015; de la Fuente, 2015; Patterson et al., 2017; Patterson & Felser, 2020). Some research suggests that clefting may increase the likelihood of attaching personal pronouns to subject antecedents (Colonna et al., 2015; Foraker & McElree, 2007), while others find no significant differences between clefted and non-clefted antecedents (Colonna et al., 2015; Järvikivi et al., 2014). In Spanish, de la Fuente (2015) found that null pronouns tended to favor non-clefted antecedents. However, research in Catalan remains limited, with the exception of de Rocafiguera (2023), who examined it-clefts and clitic-left topicalization in Catalan. On the other hand, **topicality** also influences pronoun resolution, with topicalization enhancing referent preferences (Colonna et al., 2012; de la Fuente & Hemforth, 2013). In many Romance languages, topicalization devices—particularly clitic-left dislocation (CLLD)—are central to our analysis. Mayol (2010) examined Catalan CLLD, and de la Fuente (2015) studied Spanish hanging topic left dislocations (HTLD). Both found that null subject pronouns consistently favored postverbal subject antecedents, regardless of information status or word order, and did not prefer topicalized first-mention object antecedents. In Catalan, overt pronouns showed no clear preference in CLLD OVS sentences. These findings, partially replicated by de Rocafiguera (2023), are discussed in the next section on anaphora resolution in Catalan.

Considering the evidence above, incorporating pragmatic features is crucial for disentangling syntactic functions (subject vs. object) from the linear order of mention (first vs. second) producing canonical and non-canonical sentence structures, each conveying distinct pragmatic meanings. As a result, manipulating these pragmatic features alters information structure and the sequence in which potential antecedents for subject pronouns appear. The current research thus aims to clarify how different information structures impact anaphora resolution in Catalan. **Experiments 4 and 5** incorporate non-canonical CLLD OVS word orders to specifically examine the relative weight of syntactic function (subject and object) and order of mention (first versus second), modulated by topic-focus pragmatic features. To disentangle these factors, results from these two experiments will be compared with those from Experiments 2 and 3, which used canonical SVO sentences.

To summarize, research on interpretive preferences for subject pronouns suggests that adult comprehenders strongly rely on semantic cues, such as verb meaning and gender, to disambiguate antecedents in pronoun resolution in discourse. Other cues, such as first-mention bias or subject preference, have also been shown to be powerful tools in antecedent assignment. Most results come from languages like English (and German or Finnish) that do not allow null subject pronouns in finite contexts. In the current research, we use Arnold et al. ‘s (2000) work as our starting point, aiming to replicate their experiments in a language that differs significantly in its repertoire of subject pronouns. We seek evidence on whether the privileged role of subject (or first-mention) entities, compared to other syntactic roles (e.g., objects), also applies in a language like Catalan, where the two types of subject pronouns (null and overt) are reported to exhibit a division of labor in antecedent selection.

### Anaphoric Relations Between Subject Pronouns and their Antecedents in Catalan

As a null subject language, Catalan exhibits null and overt subject pronouns whose realization and distribution are governed by discourse constraints. Broadly speaking, null subject pronouns maintain discourse topics, while overt subject pronouns express topic change. From the perspective of anaphora interpretation or resolution, the referential identification of a subject pronoun involves the selection of one antecedent in a preceding sentence (backward anaphora) or in a subsequent sentence (forward anaphora) and is also sensitive to semantic and structural cues.

One of the most influential works on anaphora resolution in null subject languages, particularly Italian, is Carminati’s (2002) research. In a series of primarily offline experiments, she proposed the *Position of Antecedent Hypothesis* (PAH), which posits a division of labor between the two types of pronouns. This hypothesis has since been tested across various languages, yielding somewhat similar results in Catalan (Bel & García-Alcaraz, 2018; Mayol & Clark, 2010) and, with some subtle differences, in other Romance and non-Romance null subject languages, such as Greek (Papadopoulou et al., 2015), Portuguese (Rinke & Flores, 2018), Spanish (Contemori & Di Domenico, 2021), and Polish (Wolna et al., 2022). According to the PAH, in a Catalan sentence like *La mare va espantar la filla quan {*pro*/ella} va entrar a la casa* (“The mother scared the little girl when {pro/she} went into the house”), the null subject pronoun *pro* in the second clause typically refers back to the subject “the mother,” while the overt pronoun ella prefers to refer to the object “the little girl” as its antecedent. With regard to Spanish anaphora resolution, a language closely related to Catalan, research has consistently shown that null pronouns display a strong subject preference, typically referring to the most prominent antecedent (usually the preceding subject). In contrast, overt pronouns exhibit greater interpretive flexibility, often signaling a topic shift or reference to a less salient antecedent. These patterns have been confirmed across different varieties of Spanish and through various methodologies (offline judgment tasks and online processing studies), underscoring the role of discourse prominence and pragmatic factors in pronoun interpretation (see Lozano, 2021, for a comprehensive overview situating these findings within broader cross-linguistic and second language acquisition research). Overall, what is common across the null-subject languages discussed here is that null pronouns consistently show a stable preference for subject antecedents, whereas overt pronouns exhibit greater variability, both across and within languages.

Few studies have examined PAH compliance in Catalan. Bel and García-Alcaraz (2018) investigated semantically ambiguous intra-sentential contexts using an acceptability judgment task and found that Catalan displays clear PAH-like biases for both null and overt pronouns, with these biases becoming more pronounced in subordinate-main clause sequences. Overt pronouns also exhibited a preference for referring to object antecedents in main-subordinate sequences. They argued that the interchangeable order of clauses entails a different organization of linguistic information, and that this aspect influences the clear compliance of the PAH, as discussed by Chamorro (2018) and de Rocafiguera and Bel (2025). Mayol and Clark (2010) also explored anaphora resolution using a two-alternative forced-choice task and a self-paced reading task. Similar to other languages, null pronouns preferred subject antecedents, while overt pronouns preferred object antecedents. However, the strength of preferences for overt subject pronouns in Catalan was weaker than in Italian, possibly due to the intersentential nature of the stimuli employed.

A remarkable study is that of de Rocafiguera (2023), who investigated the role of syntactic and pragmatic factors in pronominal subject anaphora resolution in Catalan. By manipulating information structure, she examined how syntactic function (subject vs. object), information status (focus vs. topic), and the sequential position of antecedents (first vs. second) affect the interpretation of ambiguous null and overt subject pronouns in subordinate clauses. The results from a two-alternative forced-choice task revealed that null and overt pronouns exhibit a division of labor consistent with the PAH in canonical SVO sentences, but show different sensitivities to syntactic and pragmatic cues, depending on information status manipulations in non-canonical OVS sentences. Overall, no single overriding factor—whether syntactic, pragmatic, or sequential—was identified. Null pronouns were highly sensitive to both syntactic function and information status, preferring subject and topic antecedents, with no clear specialization when these features were misaligned (e.g., subjects occupying a second-mention, postverbal position in CLLD topicalization or it-clefted focused constructions). In contrast, the interpretation of overt pronouns was primarily guided by the syntactic function of the antecedent, with a preference for object and second-mention antecedents. Unlike null pronouns, overt pronouns appeared less sensitive to the information status of their antecedents, suggesting that overt pronouns can be pragmatically inert. A subset of these results has recently been published in de Rocafiguera and Bel (2025).

Although not in Catalan, eye-tracking data has been successfully used in Spanish to shed light on pronoun interpretation and processing. Chamorro et al. (2016) conducted an eye-tracking while-reading experiment to investigate pronoun-antecedent preferences among attrited and native speakers of Spanish. Focusing on native speakers, they found that these participants showed a preference for resolving the anaphoric dependencies of null pronouns flexibly, linking them to either the subject or the object of the main clause, while overt pronouns tended to be linked to object antecedents. These results contrast with previous findings (Filiaci et al., 2014), since the flexibility for picking up antecedents was only found with null but not with overt pronouns. These differences with previous research can be due to task-dependent effects (acceptability judgment task and interpretation questions were used, respectively, as offline tasks; self-paced reading vs. eyetracking), as well as different clause order of sentence stimuli. As mentioned earlier, clausal order effects were also attested in Bel and García-Alcaraz (2015) offline interpretive data in intrasentential pronominal anaphora resolution with clearer biases in subordinate-main than in main-subordinate sentences.

As highlighted in the previous section, the accessibility information for pronoun resolution provided by the syntactic function of a potential antecedent greatly overlaps with that conveyed by the order of mention. In this sense, these two types of information are closely related and appear to remain active in the background, awaiting integration during pronoun interpretation. This helps explain the consistent findings from both approaches, as observed in non-null subject languages (see the aforementioned work by Arnold and collaborators).

In conclusion, existing evidence points to a PAH-like behavior in antecedent assignment for both null and overt pronouns in Catalan. This process has been experimentally investigated in intrasentential configurations, primarily using offline and online reading time methods. From the findings reported, it remains open to further explore how semantic and structural constraints interact in online pronoun interpretation, how and when they are accessed in real time and to what extend their integration has a different impact depending on the type of pronouns (null or overt), as previous studies have signaled in relation with other pronouns and other languages. Following Arnold et al’s (2000, 2007) work on pronoun resolution in English, which in a sense we replicate, we tested a group of Catalan speakers in order to investigate the set of issues just mentioned in intersentential anaphora contexts by means of a reference tracking experiment in a series of experiments, organized around two studies. To our knowledge, no available eye tracking data using the visual world paradigm during listening and looking at pictures exist on pronominal anaphora procedures in Catalan. The visual world paradigm has proven to be a very useful and sensitive device to investigate reference tracking (see the comprehensive volume by Knoeferle et al., 2016, for the advantages of eye movement-based studies for research on discourse and reference processing). Moreover, the fact that anaphora resolution is modulated by semantic factors such as the verb’s implicit causality and gender ambiguity makes it relevant to experimentally control for semantic effects with null and overt pronouns in Catalan. It is also necessary to provide suggestive evidence of the integration of semantic and structural information during pronominal anaphora resolution and, thus, to ascertain whether syntactic function, and/or order of mention, drives the interpretation of pronouns also at the intersentential level.

## Experiment Overview and Rationale

The current research aims to examine how semantic and word order cues are integrated in real-time pronoun interpretation, focusing on the dynamics of pronoun resolution in Catalan, a language with both null and overt subject pronouns. The experiments in this study are designed to isolate and analyze different factors influencing pronoun resolution, particularly in relation to subject versus object antecedents. Each experiment targets a distinct variable or combination of variables that may affect antecedent assignment. The study is divided into two parts: Study 1 (Experiments 1, 2, and 3) addresses the role of gender cues and pronoun type in online anaphora resolution in canonically ordered SVO sentences. Study 2 (Experiments 4 and 5) manipulates sentential order by placing the object in the first sentential position within informative, topicalized non-canonical OVS sentences, to disentangle the effects of antecedent order of mention versus their syntactic position (subject or object). As a whole, among the factors influencing subject pronoun processing, we chose gender, syntactic function, and the order of mention (i.e., information status) of antecedents.

Experiment 1 serves as a baseline investigation into the role of gender in pronoun resolution, controlling for other semantic factors, particularly implicit causality, by selecting predicates that do not favor either the subject or object antecedent. Both subject and object antecedents of different genders are presented in two-sentence stimuli, with the second sentence disambiguating the antecedent through the use of a masculine or feminine overt subject pronoun. The key prediction is that gender cues will guide antecedent resolution, with participants showing an early decision for antecedents whose gender matches that of the pronoun. It is important to underline that, in our experiments, grammatical gender and natural gender coincide, since all characters serving as potential antecedents are human, and subject pronouns in Catalan can only refer to animate referents.

Experiment 2 focuses on syntactic cues in gender-ambiguous contexts and explores whether overt subject pronouns favor one antecedent over another when both the subject and object antecedents share the same gender. The design replicates that of Experiment 1, but with the modification of one antecedent’s gender to introduce ambiguity. We hypothesize that the overt pronouns in same-gender contexts will show a possible bias towards the second-mentioned antecedent, or the syntactically less prominent object position, as often attested for null subject languages in offline experiments.

Experiment 3 extends Experiment 2 by investigating null subject pronouns, which do not provide overt gender information. By comparing the results from both experiments, we expect to find that null pronouns may exhibit a strong bias toward antecedents in subject position, different from overt pronouns, in accordance with previous offline findings.

Experiments 4 and 5 examine the role of non-canonical word order (i.e., information structure) -specifically Object-Verb-Subject (OVS) constructions with clitic left dislocations (CLLD)- to understand how syntactic function and order of mention interact in pronoun resolution. These experiments aim to disentangle the effects of syntactic function (subject vs. object) from word order (first vs. second mention). By manipulating these two factors, and comparing the results with those from Experiments 2 and 3 (which used canonical SVO sentences), we seek to identify whether the prominence provided by syntax (e.g., the subject over the object) influences pronoun interpretation more strongly than the prominence derived from discourse-pragmatics order-of-mention (i.e., topical information).

Behind these five experiments, our **hypotheses** can be summarized as follows. In Experiment 1, we predict that overt pronouns will favor gender-matching antecedents, reflecting the guiding role of gender cues in pronoun resolution. In Experiment 2, in gender-ambiguous contexts, overt pronouns may instead favor second-mentioned or syntactically less prominent object antecedents. In Experiment 3, null pronouns, lacking overt gender information, are expected to show a strong bias toward subject antecedents. In Experiments 4 and 5, we manipulate non-canonical OVS word order and topicalization, predicting that discourse-driven first mention can override canonical subject preferences.

Our study employs an eye-tracking methodology to measure these processes online, allowing us to track the fine-grained dynamics of antecedent selection. By observing when participants fixate on a particular antecedent and how quickly they resolve ambiguous pronouns, we can examine how different cues—gender, syntactic function, and order of mention—interact during pronoun resolution, providing insights into the temporal dynamics of discourse processing.

## Study 1

This study includes three experiments, each targeting a specific factor: the resolution of overt pronouns in different-gender contexts (Experiment 1), overt pronouns in ambiguous same-gender contexts (Experiment 2), and null pronouns in the same ambiguous contexts (Experiment 3). The stimuli design for all three experiments, procedure and data analysis are described collectively under Experiment 1, as the materials were lexically matched and adjusted to align with the specific experimental conditions being manipulated. This study was approved by the University Ethics Committee. Furthermore, it was conducted in accordance with the ethical standards set out in the 1964 Declaration of Helsinki and its subsequent updates (WMA, World Medical Association, 2013).

## Experiment 1

### Method

#### Data Availability

The materials, data, and scripts used in this study are available on the Open Science Framework (OSF) at the following link: https://osf.io/fup46/?view_only=18a3c7bee10e47469617c2ca9929c9c5. The dataset should be cited as follows: Bel, A., Guerra, E., Ahufinger, N., Sanz-Torrent, M., and Andreu, L. (2025, March 25). *Resolution of null and overt pronouns in Catalan.* Retrieved from https://osf.io/fup46.

#### Participants

Twelve undergraduate students from a northeast university in Spain (Catalonia) participated in the study. They spent approximately 20 min doing the experiment and received extra credit in a language course for participation. All participants were born and raised in Catalonia and their contact with Catalan started before 3. The mean age of the participants was 23.04 (range 20–33; standard deviation 3.97) and, as native speakers of Catalan, all of them self-evaluated their oral and written language skills as highly proficient. Participants provided informed consent through a form approved by the University Ethics Committee.

#### Stimuli

Experiment 1, which only included overt subject pronouns, had two independent variables, each with two levels: Gender (Masculine vs. Feminine) and Syntactic Function of the Antecedent (Subject vs. Object –or First vs. Second mention, that overlap in this experiment). Thirty-two utterances were constructed. All contained two sentences with the same structure: in the first sentence two different (or same, Exp. 2) gender characters, denoted by definite noun phrases (NPs), were included in subject and object position; a prepositional complement denoting an inanimate element depicted in the picture (filling element) ended the sentence to avoid recency effects in pronoun interpretation towards the second most recent mentioned NP- object. The second sentence began with the critical overt (or null, Exp. 3) pronoun in subject position followed by one of the two semantically empty verbs ‘portar’ (*to bring* or *to wear*) or ‘tenir’ (*to have*) that, in turn, introduced an NP-object that, together with the picture, disambiguated the interpretation of the pronoun (towards the subject or the object antecedent).

Table 1 provides examples for each condition across Experiments 1, 2, and 3, which collectively form Study 1. As said, these experiments manipulated three key variables: gender (same vs. different), antecedent (subject vs. object), and type of pronoun (overt vs. null).

**Table 1:** Example stimuli in each condition (Study 1, Exps. 1–3)

Exp	Gender Condition	Pronoun Condition	Auditory Item stimulus	Picture
1	Different gender	Overt- pronoun Subject antecedent	*La cambrera serveix el senyor a las tres en punt. Ella porta un barret mexicà perquè és la festa nacional del país.* *‘*The waitress serves the man at three o’clock. She is wearing a Mexican hat because it is the country’s national holiday.’	
1	Different gender	Overt- pronoun Object antecedent	*L’home saluda la noia un dia assolellat. Ella té una pilota als peus de les que están de moda aquest any.* ‘The man greets the girl on a sunny day. She has a ball at her feet, one of those popular this year.’	
2	Same gender	Overt pronoun- Subject Antecedent	*La cambrera serveix la senyora a les tres en punt. Ella porta un barret mexicà perquè és la festa nacional del país.* ‘The waitress serves the lady at three o’clock. She is wearing a Mexican hat because it is the country’s national holiday.’	
2	Same gender	Overt- pronoun Object antecedent	*L’home saluda el noi un dia assollellat. Ell té una pilota als peus de les que están de moda aquest any.* ‘The man greets the boy on a sunny day. He has a ball at her feet, one of those popular this year.’	
3	Same gender	Null- pronoun Subject antecedent	*El senyor persegueix el noi prop del semàfor. ___ Porta un barret perquè és carnaval i l’ocasió ho mereix.* ‘The man chases the boy close to the traffic light. (He) wears a hat because it is carnival time and the occasion merits it.’	
3	Same gender	Null- Pronoun Object antecedent	*El senyor persegueix el noi prop del semàfor. ___ Porta un barret perquè és carnaval i l’ocasió ho mereix.* ‘The man chases the boy close to the traffic light. (He) wears a hat because it is carnival time and the occasion merits it.’	

The implicit causality of the first verb was controlled in order to maintain the temporal ambiguity of the stimuli steady until the listener reached the disambiguating region (i.e. the object of the second juxtaposed sentence). Thus, all the verbs used had neutral, or almost neutral, implicit causality for assigning a subject or an object antecedent to the pronoun (bias range towards subject = 37.5%-62%). The verbs were selected based on Goikoetxea et al. (2008) normative study for Spanish, and each verb was used twice due to a low number of verbs under this condition (see Table 2 for the list of verbs employed). Controlling for all these factors allowed us to capture the interpretive preference biases of null and overt subject pronouns while referential uncertainty is still at work.

**Table 2:** Implicit causality of experimental verbs (based on Goikoetxea et al., 2008)

Verb	Bias towards subject (%)	Verb	Bias towards subject (%)
*Asustar* (to scare)	54.7	*Recoger* (to pick up)	37.5
*Corregir* (to correct)	39.2	*Saludar* (to greet)	54.3
*Enseñar* (to teach)	56.4	*Servir* (to serve)	39.2
*Entretener* (to entertain)	62.0	*Sorprender* (to surprise)	52.5
*Esperar* (to wait)	38.3	*Ver* (to see)	46.9
*Perseguir* (to chase)	37.7		

All the utterances were recorded by a female native Catalan speaker and sampled at 44,100 Hz. A digital audio editor was used to adjust each story so that the second sentence always started at 6000 ms from the first sentence onset. Utterances sounded natural and unedited to adult native speakers. This facilitated the subsequent analysis of data without having any effect on auditory stimuli. Thirty-two visual images were constructed and paired with each utterance. Each image consisted of a scene located in the center on the screen. The pictures were clip art images that were edited using an image editing software package in which appeared two characters and one distractor (see Table 1). In experiment 1, the items were balanced for the gender of the characters: half of the items contained female characters and the other half included male characters. The position of the two characters involved in each item-image (the target and the competitor, in either subject or object position of the first sentence) were presented obliquely on the computer screen and were counterbalanced and appeared equally often in the four quadrants of the screen. The audio and the visual image for each item were merged together in a video file. In each video, the onset of the spoken utterance coincided with the onset of the visual stimuli. The thirty-two experimental video stimuli created were divided into two lists, half of which were formed by trials with null pronouns and half with overt pronouns. At the end of each item, participants responded to a yes-no question assessing sentence-picture matching. All the experimental items were congruent (matching picture and auditory stimulus) since the induced anaphoric meaning was always possible in interpretive terms. Moreover, ten fillers (2 congruent and 8 non-congruent) and two congruent practice items were created and included in each list.

#### Procedure

Participants were seated approximately 22-in. in front of a Tobi T120 eye tracker with an integrated 17-in. TFT monitor. A 9-point calibration was carried out at the beginning of the experiment. The Tobii Studio software automatically validates calibrations and the experimenter could, if required, repeat the calibration process if validation was poor. Calibration took approximately 20 s. Tobii Studio software was used to present the stimuli, and collect the eye tracking data. Stimuli videos were made by images of 800 600 pixels that were represented on the screen set to 1024 768 pixels. The sounds of stimuli were presented to participants via a mono channel split to two loudspeakers positioned on either side of the viewing monitor. Participants’ eye movements were recorded while viewing the scene and listening to the utterance consisting of two juxtaposed independent sentences describing the picture. Eye position was sampled at 120 Hz (∼8-ms intervals). Participants were instructed to listen to the sentences and to judge whether the story matched the image by verbally responding “yes” or “no”. Answers were recorded in an answer sheet. There were two practice trials before the experimental task to acquaint the participant with the flow of events. Half participants were randomly assigned to one of the two lists of the experiment. The test videos were presented in random order in each list. Between each trial, participants were first presented for approximately 2000 ms with a crosshair (which they had been instructed to fixate) so that the direction of gaze on each trial would start from the samepoint (the center of the screen).

### Data Analysis

To analyze the temporal dynamics of eye movements, we employed two complementary non-parametric approaches that control for multiple comparisons and autocorrelation inherent in time-series data (Maris & Oostenveld, 2007): cluster-based permutation analysis to identify sustained time windows of differential looking, and divergence point analysis (DPA) to pinpoint the precise onset of effects with temporal uncertainty estimates (Stone et al., 2021).

#### Data Preprocessing and 95% Confidence Interval Calculation

We defined two areas of interest (AOIs) corresponding to the locations and sizes of the displayed images—the target and the competitor objects—in the visual context. The data were aligned relative to the onset of the critical linguistic stimulus (i.e., the verbal phrase in all experiments). Eye movement data were sampled at a rate of 120 Hz (~ 8 ms intervals), capturing participants’ gaze positions along the horizontal and vertical axes. Fixation data were then coded into binary values (0 or 1) based on the position of the participant’s gaze relative to the objects on the screen. Each millisecond was coded as 1 if the corresponding fixation fell within the area of interest (AOI) of the target object or the competitor object, and 0 if it fell outside these AOIs. To provide a temporal representation of visual attention, fixation proportions were calculated within 50 ms time bins to balance temporal precision with statistical stability, consistent with standard practice in visual world studies (Barr et al., 2014). All trials for every participant were processed using the R software (R Core Team, 2021). With the same software, we aggregated the eye movements data into 50-millisecond intervals for each participant, item, and specified area of interest and then computed the mean proportion of fixations and the corresponding 95% confidence intervals adjusted for within-subject designs (see Cousineau & O’Brien, 2014; Morey, 2008).

#### Cluster Analysis

Our statistical approach employed a nonparametric cluster-based permutation analysis (Barr et al., 2014; Kronmüller & Noveck, 2019; Helo et al., 2022; Guerra et al., 2024) to compare fixation proportions between the target and competitor objects under the two experimental conditions: when the agent of the action was mentioned first and when it was mentioned second. Cluster-based permutation analysis is particularly suited for time-series eye-tracking data, as it controls for family-wise error rates by accounting for temporal autocorrelation inherent in such measurements while simultaneously addressing multiple comparisons.

For each 50-ms time bin, we performed mixed-effects linear regression analyses using the lmerTest package in R (Kuznetsova et al., 2017). The dependent variable was the fixation proportion to the object (target or competitor), and the fixed effect was the object type (target vs. competitor). Random intercepts were included for participants and items to account for individual variability and item-specific effects. We identified time bins where there was a significant difference (*p* < .05) in fixation proportions between the target and competitor. Adjacent significant time bins with the same direction of effect (i.e., consistently greater fixation to either the target or the competitor) and lasting at least four consecutive bins (200 ms) were grouped together to form clusters (following Barr et al., 2014 to ensure detection of sustained rather than transient effects). For each cluster, we calculated a cluster mass statistic by summing the t-values of the individual time bins within the cluster.

To assess the statistical significance of the observed clusters, we conducted permutation tests. We generated null distributions of cluster mass statistics by randomly permuting the labels of the objects (target and competitor) within each condition across participants and trials, effectively removing any systematic differences between the two objects (Maris & Oostenveld, 2007). This permutation procedure was repeated 2,000 times for each condition. For each permutation, we identified clusters and calculated their cluster mass statistics in the same manner as with the actual data. The observed cluster mass statistics were then compared against the null distributions to obtain p-values for each cluster. Clusters were considered statistically significant if their p-values were less than 0.025 (two-tailed test), following the recommendations of Helo et al. (2022).

#### Divergence Point Analysis

In addition to the cluster analysis, we employed DPA to determine the precise temporal onset of significant differences in fixation proportions between the target and competitor objects. This method is particularly advantageous in analyzing high-resolution time-series eye-tracking data, as it addresses statistical challenges such as multiple comparisons and autocorrelation, which are common in such datasets (Stone et al., 2021, see also Fernandez et al., 2025). To identify divergence points, we applied generalized logistic mixed-effects models (GLMMs) to fixation data sampled every 50 ms. These models, incorporating random intercepts for participants and items, allowed us to compare fixation proportions between the target and competitor objects at each time point. The divergence point was defined as the earliest moment when a significant positive difference in fixation proportions for the target emerged, sustained for at least four consecutive time points (equivalent to 200 ms). This criterion ensured the detection of stable and meaningful shifts in visual attention.

Furthermore, to estimate the temporal variability of the divergence points, we implemented a non-parametric bootstrapping procedure. Bootstrap resampling was stratified by participant, item, and object type (target/competitor) to preserve the data structure. This involved resampling the original dataset with replacement to create 2,000 bootstrapped datasets. For each resample, the divergence point was recalculated, resulting in a distribution of divergence estimates. The mean and 95% confidence interval of this distribution provided robust metrics for evaluating the temporal onset of significant effects. The results of the DPA revealed the earliest time points at which participants demonstrated a preference for the target object over the competitor under each condition. For instance, when the target was mentioned first, divergence points occurred significantly earlier compared to when the target was mentioned second. These findings align with the hypothesis that linguistic cues, such as mention order, exert a measurable influence on the timing of visual attention shifts during language processing.

#### Combined Analysis

By integrating the cluster analysis and DPA, we achieved a comprehensive understanding of the temporal dynamics of the language-mediated visual attention. The cluster analysis revealed broad patterns in fixation trajectories, while the DPA pinpointed specific time points of divergence between target and competitor objects, plus an estimation of variability of that specific time point among participants. This dual approach allowed us to robustly assess the interplay between linguistic cues and visual context in our experimental paradigm, providing converging evidence for the differential processing of targets and competitors across the experimental conditions. The combination of methods provides complementary information: cluster analysis identifies sustained windows of significant effects, while DPA quantifies the precise onset with temporal uncertainty estimates, enabling direct comparison of timing differences between conditions.

## Results

**Fig. 1:**
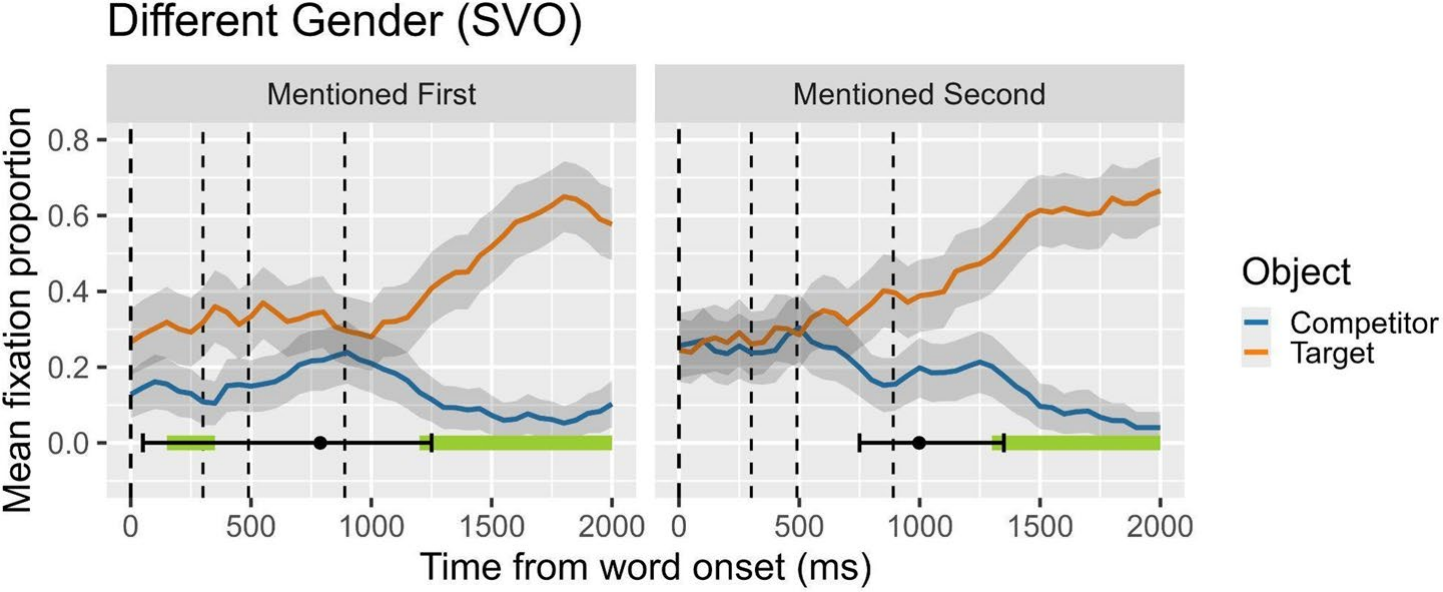
Overt pronouns in different-gender contexts. Proportion of looks to target (subject in first-mention condition; object in second-mention condition) and competitor (object in first-mention condition; subject in second-mention condition). On the x-axis, zero ms indicates the onset of the verb (second clause; first dashed line). Second dashed line marks the onset of the object complement; third and four dashed lines mark the onset and the offset, respectively, of head noun object (the disambiguating word)

Figure 1 visually presents the results of the cluster analysis and the DPI on the difference of proportion of fixations for the target and the competitor. In the figure, lines over time show the mean proportion of fixation for target and competitor, while the horizontal bars at the bottom of the plot represent the duration of clusters that reached statistical significance. As can be observed, we identified two clusters in the mentioned first condition and one in the mentioned second condition. The first two clusters extend from 150 ms to 350 ms (observed summed t = 13.64), and from 1200 ms to 2000 ms (observed summed t = 84.68) after the onset of the verb both in the mentioned first condition. The cluster for the mentioned second condition extended from 1350 ms to 2000 ms (observed summed t = 73.13), after the onset of the verb. Complementally, the DPI analysis showed in the mentioned first condition a mean onset of 787.65 ms with CI95% between 50 ms and 1250 ms. Finally, in the mentioned second condition, the DPI revealed a mean onset of 997.9 ms with CI95% between 750 ms and 1350 ms. All plots are time-locked to the onset of the verb (0ms, ‘porta’, is *wearing*, in the example in Table 1) and the subsequent vertical dashed lines mark the onset of the complement (the article ‘un’, *a*), the onset of the head of the complement (the noun ‘barret’, *hat*), and the offset of the head of the complement, *in that order*.

## Experiment 2

### Method

#### Participants

Thirteen undergraduate students from the same University context, different from those of Experiment 1, completed experiment 2, which lasted about 20 min.The mean age of the participants was 23.68 (range 20–28; standard deviation 3.68). All participants self-evaluated their oral and written language skills in Catalan as highly proficient.

#### Stimuli

Experiment 2 was designed to investigate the resolution of overt pronouns in ambiguous same-gender contexts. The effects of gender information in pronoun resolution were explored through comparison with results in Experiment 1 (non-ambiguous different-gender contexts). As in Experiment 1, the syntactic function of the antecedent (subject vs. object) or first vs. second mention was manipulated. However, in this experiment in the first sentence two same gender characters were included in subject and object position. Overt pronouns were used to express gender contrasts (*ell*, ‘he’, vs. *ella*, ‘she’). Parallel to Experiment 1, the task consisted of 32 experimental items plus 10 fillers and 2 practice items. The same construction method as in Experiment 1 was used to create the item stimuli (see previous section). Half of the overt (singular) pronouns were masculine (*ell*, ‘he’) and half were feminine (*ella*, ‘she’). The implicit causality of the first verb was controlled to prevent biases before the pronoun appeared, maintaining ambiguity until the critical region (i.e., the pronoun). Table 2 lists the verbs used along with their implicit causality bias. Examples of stimuli for each condition are shown in Table 1.

#### Procedure

The same procedure as in Experiment 1 was used.

## Results

**Fig. 2:**
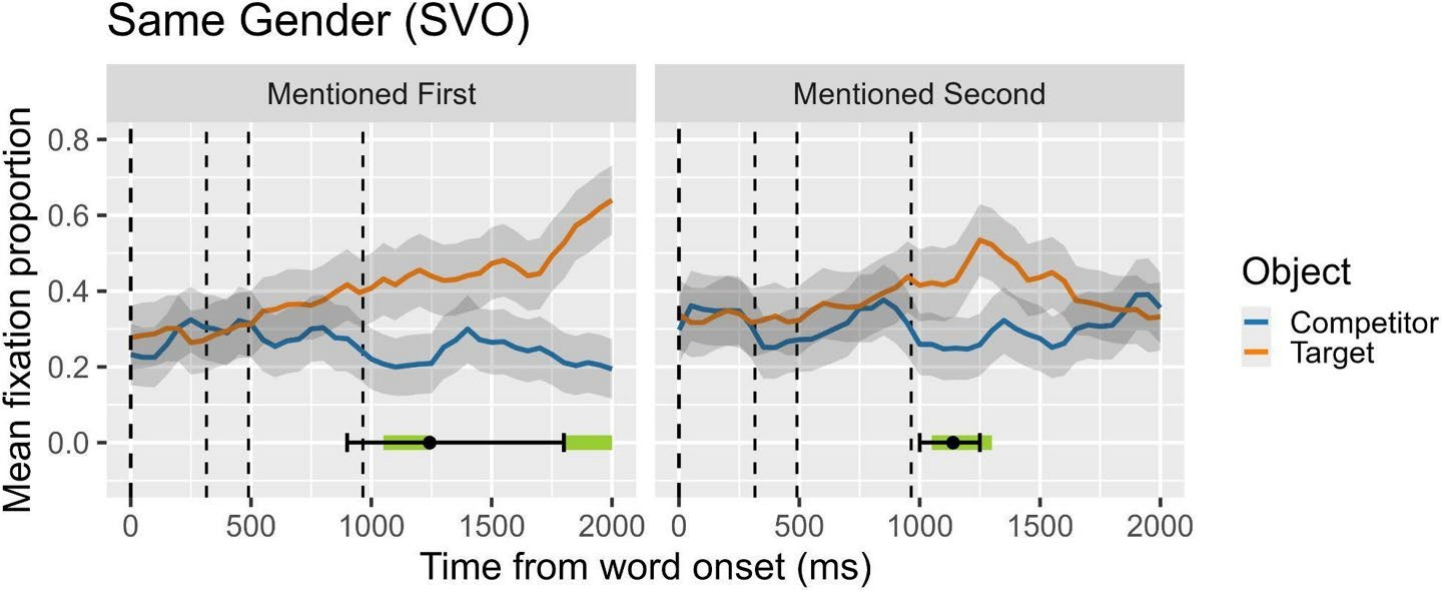
Overt pronouns in same-gender contexts. Proportion of looks to target (subject in first-mention condition; object in second-mention condition) and competitor (object in first-mention condition; subject in second-mention condition). Zero is the onset of the pronoun. Second dashed line marks the onset of the object complement; third and four dashed lines mark the onset and the offset, respectively, of head noun object (the disambiguating word)

Figure 2 visually presents the results of the cluster analysis and the DPI for Experiment 2, focusing on the difference in fixation proportions between the target and the competitor objects, and considering conditions in which the target was mentioned first or second under same-gender contexts. Similar to Experiment 1, we identified two clusters when the target was mentioned first, and one cluster when the target was mentioned second. Specifically, for the mentioned-first condition, the first two clusters extended from 1050 ms to 1250 ms (observed sum t = 16.12) and from 1800 ms to 2000 ms (observed sum t = 18.26) after the onset of the verb. In contrast, the cluster for the mentioned-second condition extended from 1050 ms to 1300 ms (t = 16.06) following the verb onset. Complementing the cluster analysis, the DPI analysis indicated that when the target was mentioned first, the mean onset of reliable divergence occurred at approximately 1242.20 ms, with a CI95% ranging from 900 ms to 1800 ms. Conversely, when the target was mentioned second, the DPI showed a mean onset of about 1138.70 ms, with a CI95% between 1000 ms and 1250 ms. As in the previous figure and the subsequent ones, all plots are time-locked to the onset of the verb, and the subsequent vertical dashed lines indicate the onset of the complement, the onset of the head of the complement, and the offset of the head of the complemen*t*, in that order.

## Experiment 3

### Method

#### Participants

In a similar vein of experiment 1, thirteen undergraduate students from the same University context, different from those of Experiment 1 and 2, completed experiment 3, which lasted about 20 min. Participants received course credit for their participation. The mean age of the participants was 22.37 (range 20–28; standard deviation 5.71). Self-assessments for oral and written comprehension and production skills indicated full knowledge of Catalan.

#### Stimuli

The same stimuli as those used in Experiment 2 were employed, but with null subject pronouns at the beginning of the second clause (see Table 1).

#### Procedure

The same procedure as in Experiment 1 was used.

## Results

**Fig. 3:**
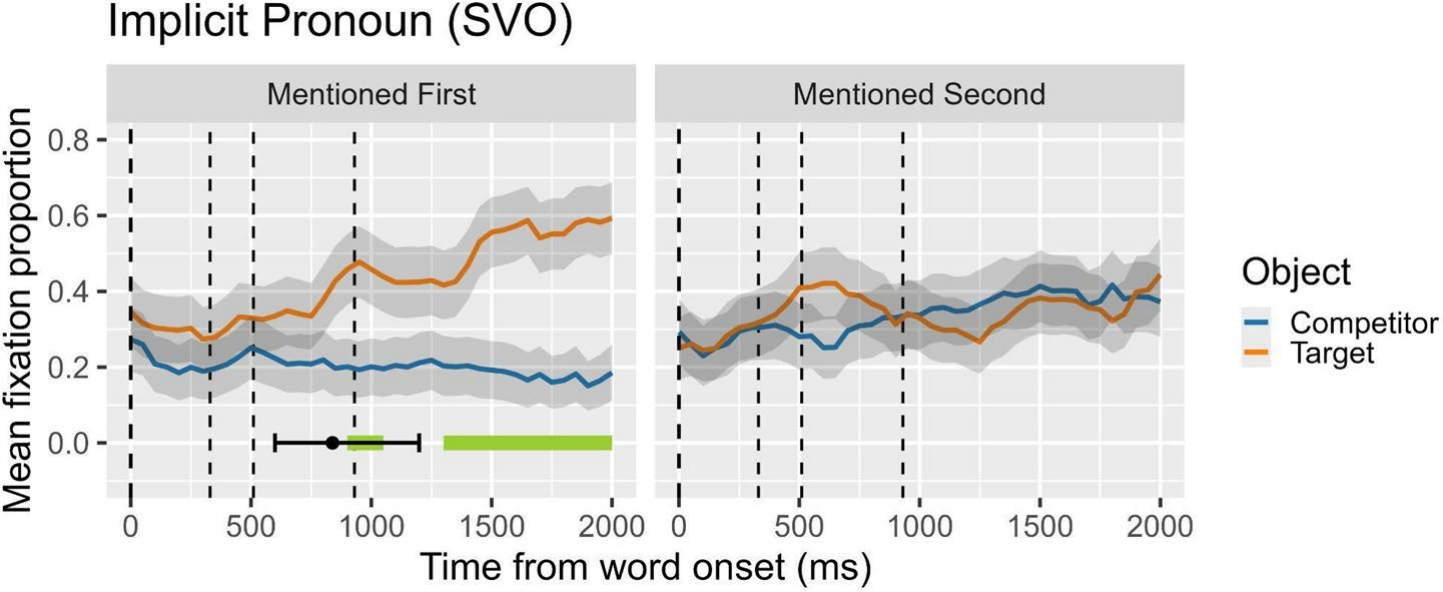
Null pronouns in same-gender contexts. Proportion of looks to target (subject in first-mention condition; object in second-mention condition) and competitor (object in first-mention condition; subject in second-mention condition). On the x-axis, 0 ms indicates the onset of the verb (the beginning of the second clause; marked by the first dashed line). Second dashed line marks the onset of the object complement; third and four dashed lines mark the onset and the offset, respectively, of head noun object (the disambiguating word)

Figure 3 visually presents the results of the cluster analysis and the DPI for Experiment 3, focusing on the difference in fixation proportions between the target and the competitor objects under implicit-gender contexts. In the figure, lines over time depict the mean fixation proportion toward the target and the competitor, and the horizontal bars at the bottom indicate clusters of statistically significant differences. Similar to previous experiments, we identified two clusters when the target was mentioned first, extending from 900 ms to 1050 ms (observed sum t = 10.47) and from 1300 ms to 2000 ms (observed sum t = 61.07) after the onset of the disambiguating word (the object head noun). In contrast, when the target was mentioned second, we did not observe any significant clusters. Complementing the cluster analysis, the DPI analysis showed that in the mentioned-first condition, the mean onset of reliable divergence occurred at approximately 838.30 ms, with a CI95% ranging from 600 ms to 1198.74 ms. For the mentioned-second condition, no reliable divergence point was detected. As with previous figures and subsequent ones, all plots are time-locked to the onset of the verb, and the subsequent vertical dashed lines indicate the onset of the complement, the onset of the head of the complement, and the offset of the head of the complement, respectively.

## Discussion of Study 1

In Study 1 we aimed to examine to what extent structural cues (antecedents in subject vs. object position) and semantic cues (different vs. same gender) guided the resolution of subject pronouns in Catalan. Additionally, we aimed to determine whether overt and null subject pronouns follow similar reference patterns or exhibit distinct intrinsic trends that set them apart in this respect.

When comparing the behavior of **overt pronouns in unambiguous contexts** (Experiment 1) **with ambiguous** contexts (Experiment 2), a different pattern emerges in online pronoun resolution, as illustrated by the contrasting resolution trajectories depicted in Figs. 1 and 2. Focusing first on non-ambiguous gender-marked contexts (see Fig. 1), participants directed their attention very early to the target referent when it occupied the first-mentioned position (i.e., the subject). This early but short-lived resolution is guided by the gender information encoded in the pronoun, suggesting that gender acted as the primary driving factor toward the first-mentioned referent during this initial segment. However, when the intended referent was in the second-mentioned position, this early decision effect was absent: in this case, the second-mentioned referents seemed to obscure or reduce the accessibility provided by the unambiguous semantic cue (i.e., gender). The Divergent Point Analysis (DPA) further highlighted a notable contrast in the timing and compactness of decision-making processes between first-mentioned and second-mentioned referents. Decisions regarding second-mentioned referents were initiated later but occurred within a more confined time frame, averaging around 1000 ms immediately following the onset of the disambiguating object noun. In contrast, decision-making for first-mentioned referents, typically occupying the subject position, exhibited greater variability in the timing of resolution. Combining insights from both analyses—CA and DPA—it is evident that gender facilitates very early attentional focus on subject antecedents but this early effect is limited to first-mentioned referents.

**Ambiguous same-gender contexts**, where gender is deactivated as an available cue, offer an optimal setting to investigate whether **overt pronouns** display an inherent resolution preference. The resolution time course illustrated in Fig. 2 reveals no clear preference for any structural position—subject or object (i.e., first or second mention)—in overt pronoun resolution. Gaze data suggest that syntactic function has little impact on interpretive biases, especially in second-mentioned contexts where resolution decisions are almost entirely absent. Consequently, this experiment failed to replicate previous offline findings that reported a bias toward object antecedents for overt pronouns in Catalan—a pattern also observed in some studies on Spanish. Instead, if a resolution decision is made, it tends to favor the first-mentioned subject antecedent. Cluster analysis (CA) provides a more nuanced view of these dynamics. For the second-mentioned object antecedent condition, a very brief significant time window (1000–1250 ms) shows more looks toward the object antecedent (the target in this condition), but this difference quickly disappears. By the end of the analyzed sequence (2000 ms, which extends beyond the disambiguating word), no resolution decision is reached, and participants exhibit a hesitative behavior. In contrast, for the first-mentioned subject antecedent condition, two discontinuous time windows reveal a pattern of resolution favoring the subject: the first window (900–1250 ms) aligns with the offset of the disambiguating word and shows significantly more looks toward the subject antecedent; this window is followed by a hesitancy period; and, finally, from 1750 ms onward, a second time window emerges, where a resolution decision favoring the subject antecedent is definitely established. In a nutshell, in the absence of semantic features such as gender, intrinsic biases of overt pronouns, if any, fail to manifest. As a result, linking a subject pronoun to an object antecedent appears to be rejected, leaving the coreferential interpretation unresolved even when the incoming information from the second sentence favors an object linkage. In summary, the comparison between Experiments 1 and 2 reveals that, when gender cues are available, they predominantly guide pronoun resolution, effectively overriding challenges associated with coreferential linking to objects. However, when gender features fail to aid antecedent identification and instead contribute redundant information (Experiment 2), their processing cost becomes unnecessary. This is consistent with findings by Gelormini-Lezama and Almor (2011) on Spanish overt pronouns, which confirmed Almor’s Informational Load Hypothesis: overt pronouns are more challenging to process unless their use serves a clear purpose.

Our findings are consistent with those of Arnold et al. (2000, 2007) for English, where participants first rely on gender information to resolve reference but, in gender-ambiguous contexts, they often default to resolving pronouns in favor of the first-mentioned referent. However, the subject preferences in our Catalan data are less pronounced. Similarly, in unambiguous, different-gender sentences, our results replicate Arnold et al.’s findings, showing that Catalan speakers effectively use gender cues to assign antecedents. Remarkably, while the anticipatory power of gender information in pronoun resolution, as posited by Arnold et al. (2000), is not strongly confirmed in our study, evidence supports the role of gender cues in facilitating coreferential linking, aligning with Hudson-D’Zmura & Tannehaus (1998) and Cunnings et al. (2017). This suggests that, while gender information is actively used to resolve pronouns, its influence may not manifest as strongly in predictive processes but rather during the integration of cues in coreferential resolution.

Nonetheless, the current findings challenge the robustness of object antecedent preferences for overt pronouns observed in offline studies in Catalan (Bel & García-Alcaraz, 2018; Mayol & Clark, 2010; de Rocafiguera, 2023), suggesting that such preferences may be task-dependent or contextually constrained. This robustness has been previously demonstrated across factors such as clause linkage and relative clause order. For instance, Bel and García-Alcaraz (2018) found that overt pronouns in Catalan were significantly linked to object antecedents in both main + subordinate and subordinate + main intrasentential clause orders. Given this robustness, one might have expected to observe a similar pattern in intersentential juxtaposed sentences, like those used in our experiments, reflecting a discourse-sustained prominence effect (Song & Fisher, 2005). However, the absence of a clear object antecedent preference for overt pronouns in our findings suggests that discourse expectations might differ between within-sentence clause relationships and between-sentence relationships. These discourse distinctions could influence the interplay between topic maintenance and topic shift, which are central to the division of labor between null and overt pronouns. We will explore this issue in more detail after discussing the results on null pronoun resolution derived from Experiment 3.

Turning our attention to **null subject pronouns** —particularly important in null-subject languages like Catalan and different from languages such as English, which only has overt pronouns in finite sentences— we can assess, by comparing data from Experiments 3 and 2, whether the division of labor between overt and null pronouns proposed in Carminati’s (2002) influential Position of Antecedent Hypothesis (PAH) extends to Catalan; specifically, we aim to determine its relevance to processing patterns in anaphora resolution. Before proceeding, let us first summarize the results from Experiment 3.

At first glance, the resolution trajectories for null pronouns in Fig. 3 closely mirror those for overt pronouns in Fig. 2, but with more distinctive and consistent resolution patterns. In the first-mention condition, an interpretive decision favoring subject antecedents is made around 720 ms on average, with an initial and sustained increase in looks toward the subject that continues to grow over time. In contrast, no decision is made in the second-mention condition, where the null subject pronoun’s referential content is linked to an object antecedent. This second option is either completely rejected or entirely disregarded. Both the CA and DPA analyses reveal a similar picture for null and overt pronouns. However, when comparing the two, the bias toward subject antecedents is stronger and emerges earlier with null pronouns (Fig. 3) than with overt pronouns (Fig. 2), although both show a clear preference for first-mentioned subject antecedents. The earlier decision-making time, reflected in the peak of looks toward the target subject—averaging 900 ms in the null pronoun experiment compared to 1250 ms in the overt pronoun experiment according to the DPA— suggests a more pronounced and consistent bias for null pronouns. It is important to remember that in both contexts, the sentences are equally ambiguous (same-gender contexts, where the gender features of overt pronouns do not aid in disambiguating interpretation, and null pronouns lack gender features entirely).

As a generalization, while both overt and null pronouns exhibit a general preference for subject antecedents, null pronouns demonstrate a more robust and faster resolution pattern. However, it remains unclear whether these differences are driven primarily by a well-defined syntactic function associated with the subject position or by pragmatic features, such as the topic feature, which is frequently linked to first-mentioned referents. This would imply that pronoun resolution may involve more intricate processing patterns in discourse-pragmatic contexts, potentially shaped by factors like the information status of elements within the discourse. While null pronouns appear to resolve reference more directly based on syntactic cues, overt pronouns may be more susceptible to a broader set of contextual influences, including syntactic position and the interpretation of prior discourse. Given this state of affairs, we propose Study 2 to explore the interaction between syntactic antecedent position and pragmatic features, particularly the role of topic realization in first-positioned elements, in shaping pronoun resolution strategies.

## Study 2

Study 2 explores non-canonical OVS word order to disentangle the effects of syntactic function (subject and object) and order of mention (first versus second) by manipulating information structure, specifically topic-focus pragmatic features, through the use of Clitic Left Dislocation (CLLD) structures. This construction places the topicalized object in the initial position and the subject in the second-mentioned postverbal position.The study includes both overt (Experiment 4) and null pronouns (Experiment 5) within same-gender ambiguous contexts. To better understand the contributions of these factors, the results will be compared with those from Experiments 2 and 3, which employed canonical SVO word orders. This study was approved by the University Ethics Committee. Furthermore, it was conducted in accordance with the ethical standards set out in the 1964 Declaration of Helsinki and its subsequent updates (WMA, World Medical Association, 2013).

## Experiment 4

### Method

#### Participants

In a similar vein of experiment 1, twelve undergraduate students from the same University context, different from those of Experiment 1, 2 and 3, completed experiment 3, which lasted about 20 min. Participants received course credit for their participation. The mean age of the participants was 21.65 (range 20–28; standard deviation 4.02). Self-assessments for oral and written comprehension and production skills indicated full knowledge of Catalan.

#### Stimuli

The stimuli in Table 3 present the topic in the first position of the sentence, marked by the grammatical marker ‘a’ for the preposed object, along with a doubled clitic (CL) within the sentence; the subject appears postverbally. These topical sentences are known as Clitic Left Dislocation (CLLD) structures. Their English translation would roughly correspond to a passive sentence: “The lady is served by the waitress at three o’clock sharp. She is wearing a Mexican hat because it is the country’s national holiday.”

**Table 3:** Example stimuli in each condition (Study 2, Exps. 4–5)

Exp	Gender Condition	Pronoun Condition	Auditory Item stimulus	Picture
4	Same gender	Overt- Pronoun Object antecedent First-mentioned	*A la senyora la serveix la cambrera a les tres en punt. Ella porta un barret mexicà perquè és la festa nacional del país.* ‘To the lady CL serves the waitress at three o’clock. She is wearing a Mexican hat because it is the country’s national holiday.’	
4	Same gender	Overt- Pronoun Subject antecedent Second-mentioned	*A la senyora la serveix la cambrera a les tres en punt. Ella porta un barret mexicà perquè és la festa nacional del país.* ‘To the lady CL serves the waitress at three o’clock. She is wearing a Mexican hat because it is the country’s national holiday.’	
5	Same gender	Null- pronoun Object antecedent First-mentioned	*Al noi el persegueix el senyor prop del semàfor. ___ Porta un barret perquè és carnaval i l’ocasió ho mereix.* ‘To the man CL chases the boy close to the traffic light. (He) wears a hat because it is carnival time and the occasion merits it.’	
5	Same gender	Null- Pronoun Subject antecedent Second-mentioned	*Al noi el persegueix el noi prop del semàfor. ___ Porta un barret perquè és carnaval i l’ocasió ho mereix.* ‘To the boy CL chases the man close to the traffic light. (He) wears a hat because it is carnival time and the occasion merits it.’	

#### Procedure

The same procedure as in Experiment 1 was used.

## Results

**Fig. 4:**
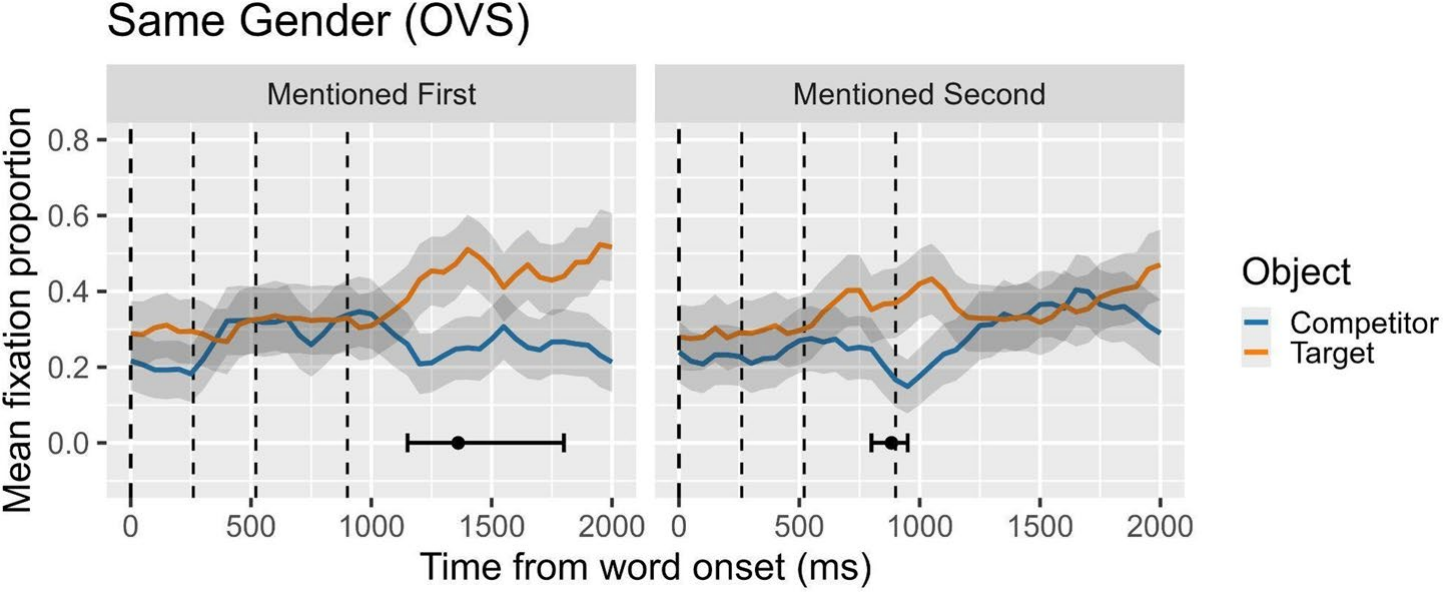
Overt pronouns in same-gender OVS contexts. Proportion of looks to target (object in first-mention condition; subject in second-mention condition) and competitor (subject in first-mention condition; object in second-mention condition). On the x-axis, 0 ms indicates the onset of the verb (second clause; marked by the first dashed line). Second dashed line marks the onset of the object complement; the third and four dashed lines mark the onset and the offset, respectively, of head noun object (the disambiguating word)

Figure 4 visually presents the results of the cluster analysis and the DPI for Experiment 4, focusing on the difference in fixation proportions between the target and the competitor objects under explicit-pronoun conditions. In the figure, lines over time illustrate the mean fixation proportion directed to the target and the competitor. Unlike the previous experiments, no statistically significant clusters of differences were observed for either the mentioned-first or the mentioned-second conditions. However, the DPI analysis indicated that when the target was mentioned first, the mean onset of reliable divergence occurred at approximately 1374.4484 ms, with a CI95% ranging from 1150 ms to 1800 ms. In contrast, when the target was mentioned second, the DPI revealed a mean onset of about 881.0065 ms, with a CI95% spanning from 800 ms to 950 ms. As with previous figures, all plots are time-locked to the onset of the verb, and the vertical dashed lines indicate the onset of the complement, the onset of the head of the complement, and the offset of the head of the complement, respectively.

## Experiment 5

### Method

#### Participants

In a similar vein of experiment 1, twelve undergraduate students from the same University context, different from those of Experiment 1, 2, 3 and 4, completed experiment 3, which lasted about 20 min. Participants received course credit for their participation. The mean age of the participants was 21.1 (range 20–28; standard deviation 1.72). Self-assessments for oral and written comprehension and production skills indicated full knowledge of Catalan.

#### Stimuli

See previous section and Table 3.

#### Procedure

The same procedure as in Experiment 1 was used.

## Results

**Fig. 5:**
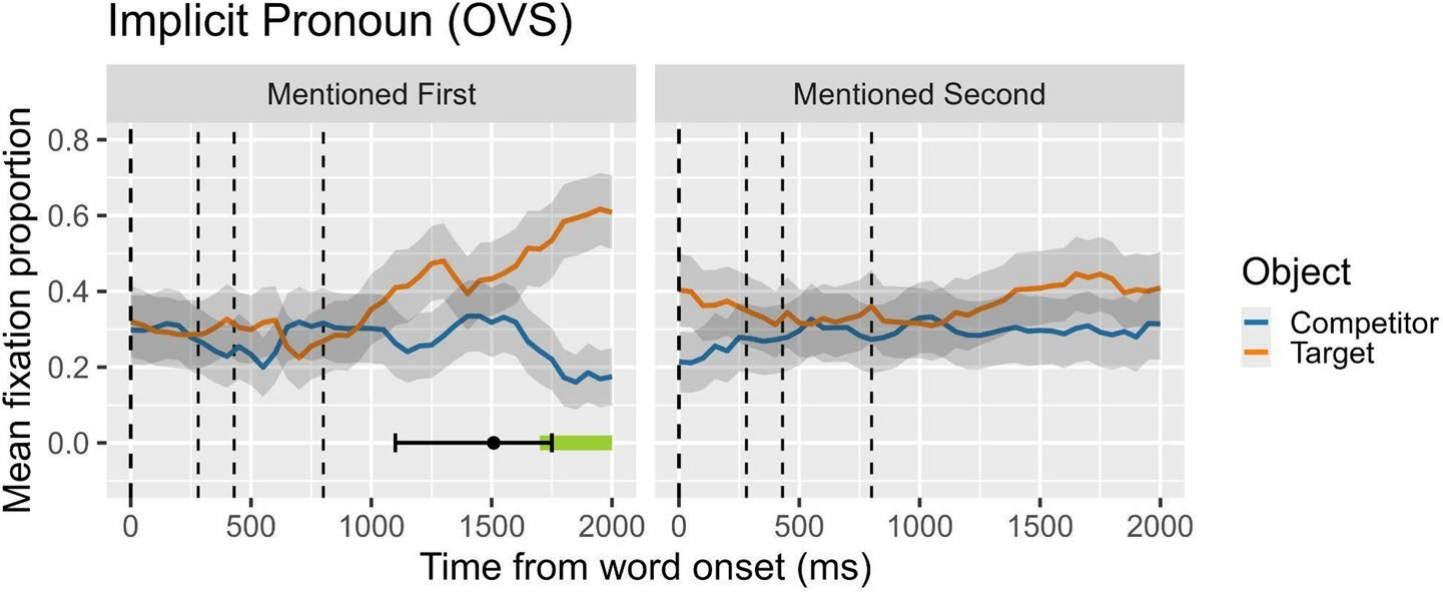
Null pronouns in same-gender OVS contexts. Proportion of looks to target (object in first-mention condition; subject in second-mention condition) and competitor (subject in first-mention condition; object in second-mention condition). On the x-axis, 0 ms indicates the onset of the verb (the beginning of the second clause; marked by the first dashed line). Second dashed line marks the onset of the object complement; the third and four dashed lines mark the onset and the offset, respectively, of head noun object (the disambiguating word)

Figure 5 visually presents the results of the cluster analysis and the DPI for Experiment 5, focusing on the difference in fixation proportions between the target and the competitor objects under implicit-pronoun conditions. In the figure, lines over time show the mean fixation proportion to the target and the competitor. The horizontal bars at the bottom indicate clusters of statistically significant differences. In this case, we identified a single significant cluster for the mentioned-first condition, extending from 1700 ms to 2000 ms (observed sum t = 29.24). For the mentioned-second condition, no statistically significant clusters were observed. Complementing the cluster analysis, the DPI analysis for the mentioned-first condition revealed a mean onset of reliable divergence at approximately 1532.20 ms, with a CI95% ranging from 1100 ms to 1750 ms. For the mentioned-second condition, no reliable divergence point was detected. As with previous figures, all plots are time-locked to the onset of the verb, and the subsequent vertical dashed lines indicate the onset of the complement, the onset of the head of the complement, and the offset of the head of the complement, respectively.

## Discussion of Study 2

Through Experiments 4 and 5, we aimed to examine the resolution of overt and null pronouns in Object-Verb-Subject (OVS) sentences, focusing on the processing of antecedents in preverbal object and postverbal subject positions. The primary research questions addressed how syntactic function and linear order influence pronoun resolution, particularly in non-canonical word orders, and whether pronoun type (null vs. overt) affects processing efficiency. Specifically, we investigate whether the first-mention position enhances antecedent accessibility and whether the subject preference observed in SVO structures persists when subjects occupy the second position in OVS sentences.

Focusing first on overt pronouns, Fig. 4 shows that the proportion of looks to the target increases over time (left panel) when the object—but not the subject—occupies the first position in the sentence. However, without clearly identifying clusters to distinguish the target (the first-positioned object) from the competitor (the second-positioned subject), the supposedly privileged first position loses its strength as a coreferential link for an overt pronoun. This suggests that when the grammatical subject and the sentence-initial position are not aligned, the prominence of the first position by itself is diminished. The Divergent Point Analysis (DPA) further indicates that participants resolve pronoun ambiguity relatively late, suggesting delayed processing when the antecedent appears in the preverbal object position—a less canonical and potentially more cognitively taxing structure.

In contrast, for antecedents in the postverbal subject position (right panel), no clusters are identified either. However, the DPA reveals an earlier resolution in the time course compared to the first-mentioned condition, albeit over a brief time span. This first-step, faster decision reflects a general preference for subjects as antecedents (even when they appear in the second position). Nevertheless, because postverbal subjects occupy a non-canonical position, the divergence between the target and competitor diminishes rapidly, resulting in indecision or unresolved coreference, as shown by the overlap of the orange and blue lines.

In a nutshell, when sentence position and grammatical function are misaligned in the context of atypical word order (OVS), the first position attenuates its privileged status, and the subject (in second position) is obscured and disadvantaged to establish a coreferential link.

Turning our attention to the resolution of null pronouns in OVS sentences (Fig. 5), the results mostly replicate those reported for overt pronouns, though with more pronounced contrasts. Fixation data from the first-mentioned condition (left panel) show that resolution (inferred from the divergence of the orange and blue lines) mirrors that of overt pronouns in the corresponding condition (Fig. 4). Specifically, pronoun interpretation is directed toward the first-mentioned object, with DPA values indicating resolution and significant differences emerging in the CA from 1700 ms onward, a delayed time of resolution corresponding to one word after the disambiguating word. This is marked by a gradual increase in fixations on the object antecedent. Comparing this with the DPA from Fig. 4 for overt pronouns—while not a direct or entirely straightforward comparison—suggests that the DPA for null pronouns occurs slightly later. This delay points to higher cognitive costs associated with resolving null pronouns when the antecedent is an object, despite the object occupying the first position in the sentence.

As for the second-mentioned postverbal subject antecedents, fixation data show no resolution of the null pronoun whatsoever: attention remains distributed, without a clear shift towards the target even after the disambiguating word is reached.

In summary, for null pronouns in OVS sentences, the first position retains its prominence as the preferred locus for the antecedent (in this case, the syntactic object). In contrast, the second position, despite containing the syntactic subject, becomes coreferentially opaque for a null pronoun.

In conclusion, the resolution of null and overt pronouns in OVS sentences highlights the dominant influence of sentence position over syntactic function. While object antecedents in their canonical postverbal position in SVO sentences did not establish a clear resolution dependency with either overt or null pronouns (Exps 2 and 3), preverbal object antecedents in the first position facilitate resolution for both pronoun types (Exps 4 and 5). However, null pronouns incurred higher cognitive costs and delayed fixation onset. In contrast, postverbal subject antecedents, despite the typical subject preference in SVO contexts, fail to elicit a clear resolution for either pronoun type in OVS structures. These findings show that positional cues play a bigger role than grammatical subjecthood in resolving pronoun ambiguity, as critically demonstrated in non-canonical word order.

## General Discussion

In this study, we explore a long-standing debate on the factors influencing subject pronoun resolution, with a particular focus on Catalan, a null-subject language. We investigate linguistic factors such as gender, pronoun type, and the syntactic function of the antecedent (subject or object), along with a cognitive strategy—namely, the order in which antecedents appear (first- or second-mentioned position).

Through Experiments 1, 2, and 3 (Study 1), we examined the role of gender and pronoun type in pronominal resolution within SVO canonical order sentences. Our results demonstrate that gender cues facilitate early resolution but interact with antecedent position, primarily influencing first-mentioned referents. Pronoun type also plays a critical role: while null pronouns consistently prefer subject antecedents -aligning with prior offline and online findings- overt pronouns deviate from previous offline studies, favoring first-mentioned subjects but showing no preference for objects. Overall, Study 1 highlights key patterns in Catalan pronoun resolution. First, across both unambiguous and ambiguous contexts, there is a clear preference for first-mentioned subject antecedents, especially when gender cues are available (both grammatical and natural, as they coincide in our experimental design), reinforcing the central role of syntactic function in guiding overt pronoun resolution. In unambiguous gender-marked contexts, overt pronouns rely heavily on gender cues to resolve reference, displaying a clear but individually variable bias toward the first-mentioned subject antecedent. However, in ambiguous, same-gender contexts, this reliance on gender cues dissipates, and overt pronouns show a more hesitant and delayed resolution pattern to the first-mentioned subject, and fail to show a preference for either subject or object when the disambiguating region supports the second-mentioned object. In contrast, null pronouns show a consistent and robust preference for subject antecedents with resolution decisions occurring significantly earlier than for overt pronouns. This difference may be related to the more limited morphological information carried by null pronouns, which makes their consistent pairing with an antecedent more critical. However, the results from Experiment 2, where gender distinctions were neutralized for overt pronouns, suggest that the stronger biases of null pronouns instead reflect their inherently stronger antecedent preferences, together with the greater referential flexibility observed for overt pronouns. In sum, the complementarity and division of labor between null and overt pronouns—where the former favor first-mentioned subject antecedents and the latter prefer second-mentioned object antecedents, as proposed by the PAH and supported by previous studies—are not overtly reflected in our gaze data. We elaborate on this issue further below.

The stronger attention that subject antecedents attract from null pronouns, as revealed by our eye-movement data, confirms findings from previous studies in Catalan (Bel & García-Alcaraz, 2018; Mayol & Clark, 2010; de Rocafiguera, 2023; de Rocafiguera & Bel, 2025) and in other null-subject languages such as Spanish (Contemori & Di Domenico, 2021; Filiaci et al., 2014). This bias is consistently observed across diverse methodologies, including online measures (e.g., reaction times, eye-tracking) and offline tasks (e.g., interpretation and production), and persists across various discourse contexts, encompassing both intrasentential and intersentential settings, as examined here. These findings underscore the robustness of null pronouns in encoding discourse-pragmatic features that predominantly signal reference maintenance. As a result, null pronouns exhibit a strong tendency to refer back to elements that function as subjects or topics in preceding sentences, clauses, or broader discourse contexts.

In our investigation in Study 2 of online pronoun resolution over time in OVS sentences Experiments 4 and 5), we found that sentence position plays a slightly more prominent role than grammatical subjecthood for both null and overt pronouns. It is important to highlight the work on Catalan pronominal resolution by Rocafiguera (2023) and Bel & Rocafiguera (2025), as our findings align with and extend their research. They studied the interplay between syntactic function and information structure in pronoun resolution and suggest that non-canonical word orders can obscure or reduce the resolution biases typically observed in canonical structures. Specifically, both studies confirm that pronoun resolution is influenced not only by syntactic function but also by the information status of antecedents. This conclusion holds true in our current study despite differences in data origins: offline vs. online methods.

Rocafiguera (2023) and Bel and de Rocafiguera (2025) found that Catalan null and overt pronouns exhibit a division of labor consistent with Carminatti’s (2002) Position of Antecedent Hypothesis (PAH). Specifically, in canonical SVO sentences, null pronouns tended to prefer subject antecedents, while overt pronouns favored object antecedents. However, our study did not replicate this pattern. Instead, both null and overt Catalan pronouns in ambiguous contexts showed a preference for the first-position subject in SVO sentences (Experiments 2 and 3), mirroring findings for English overt pronouns in Arnold et al. (2000, 2018). Crucially, this subject preference weakened when the subject antecedent appeared in a non-canonical postverbal position in OVS sentences. This attenuation of subject prominence in coreference aligns with Bel and de Rocafiguera (2025), who found that manipulating word order through CLLD structures (resulting in OVS order) disrupted pronoun biases: null pronouns lost their preference for subjects, while overt pronouns lost their preference for objects, often remaining neutral or failing to resolve toward either antecedent. The authors suggest that while null pronouns are highly sensitive to syntactic function and information structure, overt pronouns rely more on the hierarchical position or linear order of the antecedent. This claim aligns specifically with Kaiser and Trueswell’s (2008) form-specific approach to anaphora resolution, which indicates that different pronouns (including anaphoric demonstratives, see Hert et al., 2024 for German demonstratives compared to subject pronouns) are sensitive to distinct constraints. Overall, our results underscore that in non-canonical OVS structures, positional cues—particularly first position—play a stronger role in pronoun resolution than syntactic subjecthood: as sentence structure deviates from the canonical SVO order, the influence of subjecthood diminishes, reinforcing the importance of positional factors in pronoun interpretation.

This outcome -the prominence of sequencing over subjecthood- does not align with recent findings on the German pronoun *er* and the demonstrative *der* by Hert et al. (2024), who examined relative accessibility, or prominence, by manipulating sentential word order in focused sentences. Their gaze data demonstrated that subjecthood overruled order-of-mention and information structure. The differences can be attributed to variations in the experimental setup, as they manipulated order using prosodic cues and larger preambles to mark focused, preposed referents. In our case, with CLLD structures, the preposed antecedents do not convey a focus feature but rather a topic feature, which carries distinct pragmatic value. In line with Centering Theory, topicalized sentences tend to establish a discourse configuration in which topicality, rather than syntactic function, determines referential continuity. Since topicality governs the discourse prominence and accessibility of referents beyond their grammatical roles, the order of mention becomes a key factor guiding interpretation. Consequently, differences along the pragmatic dimension—specifically those related to topicality—may lead to differential preferences regarding syntactic function and linearization. Thus, the order-of-mention effects observed in Experiments 4–5 likely reflect discourse-pragmatic constraints associated with topicalization, rather than being driven solely by syntactic factors. Unlike in Carreiras et al. (1993), where order of mention primarily reflected the linear sequence of subjects and objects, in our experiments it is tied to informational structure, pragmatically determined by topicalization. Our findings therefore extend those of Carreiras et al., showing that discourse-driven ordering, rather than purely syntactic sequencing, modulates referential preferences in Catalan. In short, linear order alone does not determine the preferences of null and overt anaphors; rather, these preferences reflect the effects of linearization shaped by discourse-pragmatic factors and, to a lesser extent, by syntactic function. This aligns with accessibility-based accounts of anaphor resolution (Ariel, 1990; von Heusinger & Schumacher, 2019), which propose that more prominent or salient referents are more easily retrieved during processing. Consistent with Van Berkum et al. (2007), who show that pronoun resolution is incremental and sensitive to discourse prominence, our results suggest that referential accessibility (driven by discourse-prominent topicalized constituents rather than purely syntactic subject–object order) guides pronoun resolution in Catalan, with syntactic function playing a secondary role.

In addition, findings from de Rocafiguera (2023) and de Rocafiguera and Bel (2025) on Catalan focused elements expressed through it-clefts partially overlap with those reported here. Antecedents in preposed left-peripheral positions (object clefts) retained their potential as antecedents—particularly for null pronouns—showing effects of linearization (first position) over subjecthood, contrary to the “anti-focus effect” reported by Patterson and Felser (2020). By contrast, in CLLD structures, null pronouns lost their preference for subject antecedents and remained neutral. Overt pronouns in SVO sentences preferred object antecedents, differing from our results, and showed no clear preference in CLLD or object-cleft configurations. These findings, however, are based on offline data reflecting final interpretation rather than online resolution, and therefore do not indicate a processing bias for subject pronouns. Of course, it remains uncertain whether introducing larger preambles to our stimuli would more effectively identify the topic of the sentences, which is particularly relevant in pragmatically marked constructions such as CLLD. In our defense, we should note that the displaced object, which conveys a pragmatic topic, is grammatically marked with the preposition ‘a,’ indicating that it functions as an object and cannot be confused with a subject, which, according to general patterns in sentence processing, is typically expected to appear in first position (Mahowald et al., 2023). Finally, we want to emphasize that while position appears to play a more prominent role, it does not negate subjecthood as a cue for pronoun resolution, in line with the convergent approach proposed by Gordon and Chan (1995). Position seems to take precedence only when the two cues are misaligned.

The observed differences in the relative prominence of cues -whether position or subjecthood plays a stronger role- can be attributed to language-specific properties. In languages like German and Finnish, where pronouns are always overt, subjecthood is the dominant cue for pronoun resolution, as there is no contrast between null and overt forms to signal referential shifts: subject continuity thus tends to be the default interpretation (Song & Fisher, 2005), with pronouns resolved based on syntactic function rather than position. In contrast, in null-subject languages like Catalan and, likely, Spanish, the presence of both null and overt pronouns introduces a distinction in referential preferences: null pronouns typically refer to the most prominent antecedent, usually the subject, while overt pronouns are more likely to signal a reference shift (Carminati, 2002). This contrast makes positional prominence, especially first-mention effects, a stronger determinant in pronoun resolution. Thus, while subjecthood is the primary cue in languages with only overt pronouns, positional factors and order-of-mention play a more significant role in null-subject languages, where pronoun form also contributes to referential resolution.

There is still an interesting finding that deserves further attention. We did not identify an inherent preference associated with either pronoun under any conditions. If null or overt pronouns had an intrinsic bias, we would expect to see it emerge from the moment the pronoun or verb is encountered until just before the disambiguating word appears, specifically, between 0 ms (the verb’s onset in the figures) and approximately 500 ms (marking the onset of the disambiguating word). Eye-tracking data from this time window can provide insight into processing before disambiguation occurs. An examination of all figures reveals no region where one antecedent (the target or the competitor) attracted more visual attention. The only exception is in Experiment 1, where, in the different-gender condition, a brief early preference was detected but quickly abandoned. Notably, even the unambiguous gender cue (*él/ella*, ‘he/she’), which clearly signals the matching antecedent, was not strong enough to establish and maintain a resolution decision. This suggests that pronoun resolution is not finalized upon encountering the pronoun (or verb) but remains an ongoing process. Our results are somewhat mixed, reflecting previous literature: while Hudson-D’Zmura & Tanenhaus (1998) and Cunnings et al. (2017) argue that gender cues have an early impact on antecedent-pronoun linking, Garnham et al. (1992) and McDonald and MacWhinney (1995) suggest their influence is limited, short-lived, or not consistently maintained in anaphoric processing.

## Limitations and Future Directions

This study highlights the complex interplay between syntactic function, positional cues, and pronoun type in Catalan pronoun resolution, but several limitations remain. First, the relatively small number of participants per experiment (around 12–13). Although this sample size aligns with standard practice in eye-tracking research, it may still constrain the generalizability of the findings. Importantly, the analytic approach used here—combining linear mixed-effects modeling, cluster-based permutation testing, and divergence point analysis (DPA)—maximizes the information extracted from the time-course data by accounting for both subject- and item-level variance and by identifying statistically robust effects across consecutive time windows. These techniques mitigate the impact of smaller sample sizes by capitalizing on the rich, high-density nature of eye-tracking data. Nevertheless, future research with larger and more diverse samples would further validate these results and allow for a more detailed examination of individual variability in pronoun resolution processes. Second, the divergence from previous offline studies suggests that processing and final interpretation may rely on distinct mechanisms, requiring further research that integrates both online and offline methodologies. Third, the findings raise questions about the role of discourse-pragmatic features, particularly in non-canonical topical structures like CLLD, which should be explored using additional experimental conditions (e.g., topical and focused sentences via it-clefts) and broader contextual manipulations. Including larger contexts, such as preambles to our sentence stimuli, would help clarify the contribution of pragmatic roles —particularly topic— in both topic maintenance and topic shift contexts (i.e., first- or second-mentioned antecedents), as well as their influence on the preferences that null and overt subject pronouns exhibit in Catalan. Fourth, the absence of an early intrinsic preference for either pronoun type before disambiguation suggests that pronoun resolution is more dynamic than previously assumed, warranting finer-grained temporal analyses. Finally, future research should investigate cross-linguistic differences in cue prominence, particularly in other null-subject languages, such as Spanish (as well as null-object languages, such as Mandarin Chinese), to better understand the interaction between subjecthood and linear position in pronoun resolution.

## Supplementary Information

Below is the link to the electronic supplementary material.

Supplementary file1 (DOCX 15 KB)
