# Supplementary material for: Resolution of Null and Overt Pronouns in Catalan: An Eye Tracking Study
Source: J Psycholinguist Res. 2026 Jan 10;55(1):7. doi: 10.1007/s10936-025-10189-7 (PMC12790527; doi:10.1007/s10936-025-10189-7)
Supplement: Supplementary file 1 — Supplementary file1 (DOCX 15 KB) [file 10936_2025_10189_MOESM1_ESM.docx]

Supporting data

The materials, data, and scripts used in this study are available on the Open Science Framework (OSF) at the following link:<https://osf.io/fup46/?view_only=18a3c7bee10e47469617c2ca9929c9c5>.
